# Assessment of China’s H5N1 routine vaccination strategy

**DOI:** 10.1038/srep46441

**Published:** 2017-04-19

**Authors:** Zhen Sun, Jimin Wang, Zeying Huang

**Affiliations:** 1 Institute of Agricultural Economics and Development, Chinese Academy of Agricultural Sciences, Beijing 100081, China; 2 School of Management, Inner Mongolia University of Technology, Hohhot 010051, China

## Abstract

In this study, a simulation model was used to assess the epidemiological and economic impacts of the routine vaccination strategy for H5N1 in China at the national level. The results of the simulation show that a routine vaccination strategy at the national level could have a substantial impact on decreasing H5N1 outbreaks; it could therefore markedly reduce the severity and duration of an H5N1 epidemic at the national level in China. Under a routine vaccination programme, when a flock is infected, the number of depopulated birds could be reduced by approximately 91%, the outbreak duration could be reduced by one-fourth, and the probability of an H5N1 outbreak could be reduced to 51.5%. Although the use of vaccination has obvious benefits, if indirect costs were not considered, the mean direct cost of simulated disease control without vaccination was only 1.36% of the cost of the routine vaccination strategy, and this former approach would have saved 57 billion yuan for all poultry in China from 2004 to 2012. Traditional H5N1 control strategies with culling programmes at the national level represented a better choice for China.

H5N1 is a highly pathogenic avian influenza (HPAI) that began with a single report of mild disease in geese in Guangdong, China, in 1996 but has since spread to cause infections and high death rates in poultry and wild birds in 63 countries. H5N1 HPAI has a profound impact on animal welfare and the poultry industry in China. From 2004 to 2013, more than 110 outbreaks in domestic poultry occurred in 23 provinces and caused severe economic damage to the poultry industry in China. Since 2004, over 36 million poultry have been depopulated to control the disease. H5N1 HPAI is also considered a serious threat to humans. Since the first recorded direct bird-to-human transmission of H5N1 HPAI in Hong Kong in 1997, the World Health Organization (WHO) has recorded 52 confirmed H5N1 cases with 31 fatalities in China, representing a 60% case fatality rate.

China is one of the largest producers of poultry worldwide, with a production of some 18 million tonnes of annual poultry meat output, which accounted for 18% of global production in 2013. The poultry inventory in China is 5.8 billion birds, and the annual output value from poultry production in China has been estimated at 660 billion yuan^1^. China has a high poultry density and a very large number of small farmers; thus, the spread of H5N1 avian influenza in China is markedly different from that in the developed poultry sector in the west. In 2010, there was an inventory of 324.39 layers per km^2^ and 1143.3 broilers for slaughter per km^2^ in China. Although large producers dominate the poultry sector, the traditional system of small producers still prevailed. In 2010 in China, there were 20.64 million layer farms with inventories of 499 or fewer birds. These farms account for 96.85% of all layer farms, which have a mean of 27.91 birds per farm; the layer inventory of these farms is 21.19% of the total layer inventory in China. In 2010, there were 24.83 million broiler farms raising poultry operating at a scale below 2000 birds for slaughter. These farms accounted for 97.99% of all broiler farms, and the average bird slaughter of these farms was 55.08 birds, which provided 14.27% of broiler production.

Traditionally, HPAI control strategies have used various components, including rapid diagnostics and surveillance, elimination of infected flocks, enhanced biosecurity, and education/training of poultry workers^2^. When necessary, the most common additional measure is pre-emptive culling around infected flocks^3^. Vaccination for H5N1 HPAI was first implemented in 2002 in Hong Kong. From 2002 to 2010, more than 113 billion doses of the avian influenza vaccine were used in at-risk national poultry populations that accounted for more than 131 billion birds in 15 countries. The average national vaccination coverage rate was 41.9%, and the global avian influenza vaccine coverage rate was 10.9% for the 15 vaccination countries. Most of these vaccines were used in the H5N1 HPAI panzootic, and more than 99% were employed in four countries: China (90.99%), Egypt (4.65%), Indonesia (2.32%) and Vietnam (1.43%)^4^.

In China, a culling strategy with routine vaccination is used to control H5N1 HPAI outbreaks at present. After final confirmation of an H5N1 infection, all of the poultry within a 3-km radius are depopulated. China initiated an emergency vaccination strategy in 2004 in which only birds in the buffer zone were required to be vaccinated. However, in late 2005, this strategy was changed to a routine (mass) vaccination strategy. The government provided financial support for 100% vaccination coverage of domestic poultry^5^. The annual H5 AI vaccine usage was higher in China than in all other countries, with between 18.9 and 23.7 billion doses used per year from 2006 to 2009^4^. In 2008, the estimated H5N1 HPAI vaccine and vaccination costs of Chinese poultry were more than 6.2 billion yuan. From 2011 to 2020, the H5N1 HPAI vaccine and vaccination costs of Chinese poultry will reach 75.2 billion yuan^6^.

Over the last few decades, stochastic simulation models have been used to examine the consequences of animal disease outbreaks and analyse various control measures^7^,^8^,^9^. Epidemiologic simulation modelling of HPAI outbreaks provides a useful conceptual framework with which to estimate the consequences of HPAI outbreaks and evaluate disease control strategies^10^,^11^. However, most simulation models for the spread and control of animal diseases have been conducted at the regional level^11^.

The aim of this study was to assess the epidemiological and economic impacts of the routine vaccination strategy for H5N1 HPAI in China at the national level. To achieve this aim, an epidemiological simulation model was used to evaluate H5N1 avian influenza outbreak scenarios in China by employing a culling strategy with routine vaccination for disease control. An alternative culling strategy without vaccination was also evaluated. Because the indirect costs of the Chinese H5N1 epidemic were not available, only estimates of the direct costs were calculated in the model; indirect costs were not considered in this study.

## Results

### Culling strategy with routine vaccination (baseline)

**Figure 1:**
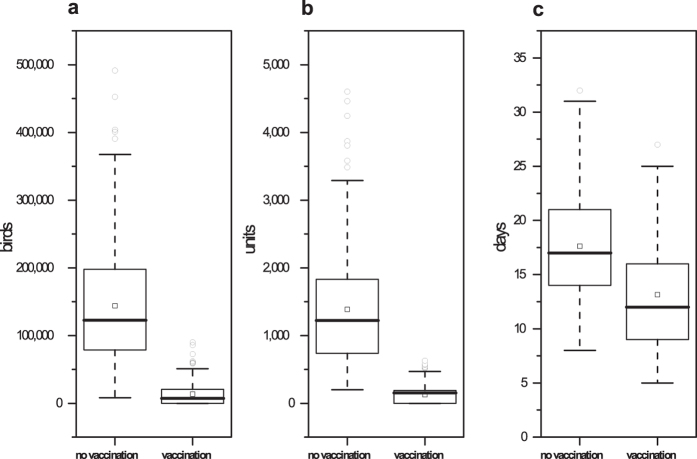
Epidemiological outcomes in culling strategies with and without routine vaccination. The boxplots represent the number of depopulated birds, the number of depopulated units and the outbreak duration for each strategy (no vaccination or vaccination). The squares (◽) represent the means under different conditions.

In this study, an outbreak means that H5N1 clinical signs are observed and reported to the local government. There were 103 outbreaks in 200 random simulations when a flock was infected at the national level in China based on routine vaccination strategies. Thus, the probability of an H5N1 HPAI outbreak is 51.5%. As shown in Fig. 1, a mean of 13,432 birds and 124.49 units were depopulated; the mean outbreak duration was 13.15 days in each simulated outbreak.

### Sensitivity of the results from the culling strategy with routine vaccination to changes in the unit density

**Figure 2:**
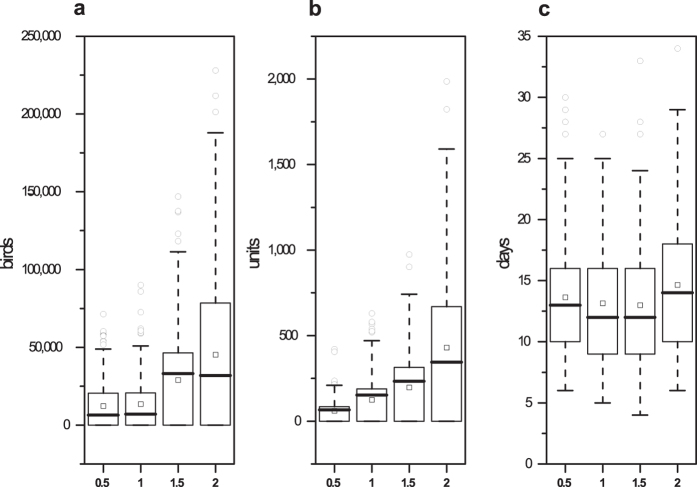
Results of the sensitivity analysis of changes in the flock density. The boxplots represent the number of depopulated birds, number of depopulated units and outbreak duration with 0.5-, 1-, 1.5- and 2-fold of the average unit density. The squares (◽) represent the means under different conditions.

Higher flock densities resulted in a greater frequency of outbreak development, higher numbers of depopulated birds and longer outbreak durations. We explored the sensitivity of the model to changes in the unit density by varying the unit density by 0.5-, 1.5- and 2-fold the average (baseline). The results of 200 simulations are shown in Fig. 2. When the density of the flocks was changed from 0.5- to 2-fold (300% increase), the number of depopulated birds changed from 12,320 to 45,150 (266.48% increase), the number of depopulated flocks changed from 60.88 units to 430.13 units (606.52% increase), and the outbreak duration changed from 13.63 days to 14.66 days (7.56% increase). Moreover, the number of outbreaks changed from 111 to 120 (8.11% increase) in 200 random simulations.

### Sensitivity of the results from the culling strategy with routine vaccination to changes in the unit size

**Figure 3:**
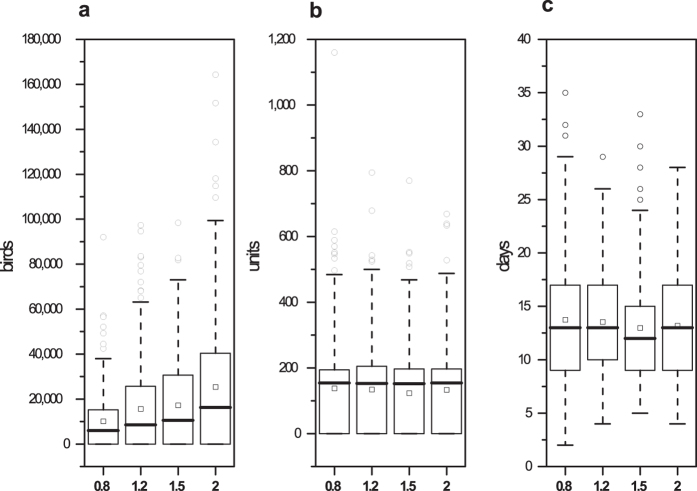
Results of the sensitivity analysis of changes in the unit size. The boxplots represent the number of depopulated birds, number of depopulated units and outbreak duration with 0.8-, 1.2-, 1.5- and 2-fold of the average unit size. The squares (◽) represent the means under different conditions.

When the unit size was increased, the number of depopulated birds exhibited a similar proportional change. When the flocks were depopulated, the outbreak duration and total outbreaks did not significantly change. Suppose every unit size is changed to 0.8-, 1.2-, 1.5- and 2-fold of the average unit size. The results of 200 simulations are shown in Fig. 3. When the unit size changed from 0.8- to 2-fold (150% increase), the mean number of depopulated chickens changed from 10,125 to 25,365 (150.52% increase), the number of depopulated flocks changed from 137.85 units to 133.32 units (3.29% decrease), and the outbreak duration changed from 13.74 days to 13.17 days (4.15% decrease). Moreover, the number of outbreaks changed from 111 to 109 (1.8% decrease) in 200 random simulations.

### Culling strategy without vaccination (baseline)

As shown in Fig. 1, a mean of 144,040 birds were depopulated, a mean of 1,385.54 units were depopulated, and the mean outbreak duration was 17.63 days. The total number of outbreaks was 200 in 200 iterations. This result indicates that the probability of an H5N1 HPAI outbreak is 100% when a flock is infected in culling strategies without vaccination.

### Sensitivity of the results from the culling strategy without routine vaccination to changes in the probability parameters for observing clinical signs

If the probability of observing clinical signs was cut in half on the first two days, compared with the baseline, the number of depopulated birds increased by 21.01%, the number of depopulated flocks increased by 21.65%, and the outbreak duration increased by 17.47% in 200 simulations. These results indicate that rapid diagnoses are important to combat the threat of H5N1 HPAI.

### Sensitivity of the results from the culling strategy without routine vaccination to changes in the contact rates

**Figure 4:**
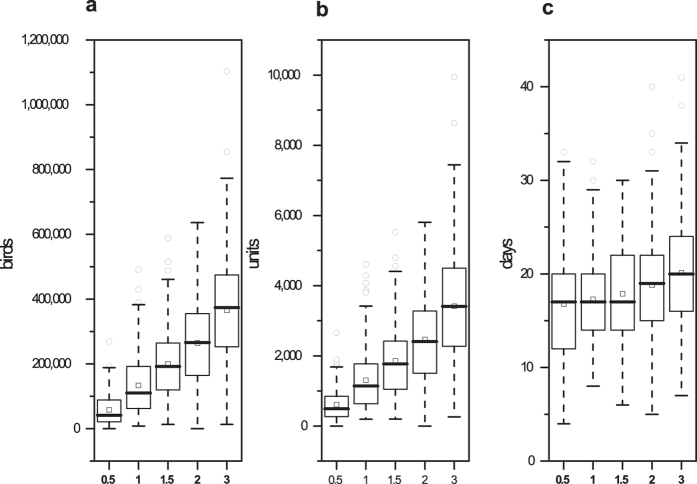
Results of the sensitivity analysis of changes in the contact rates. The boxplots represent the number of depopulated birds, the number of depopulated units and the outbreak duration with 0.5-, 1, 1.5-, 2- and 3- fold of the contact rates. The squares (◽) represent the means under different conditions.

The contact rate has a notable impact on the epidemic severity. A higher contact rate indicates a more serious epidemic. Suppose the contact rates were changed to 0.5-, 1.5-, 2- and 3-fold of the baseline. The results of 200 simulations are shown in Fig. 4. When the contact rates were changed from 0.5- to 3-fold (500% increase), the average number of depopulated chickens changed from 58,233 to 365,523 (527.69% increase), the number of depopulated flocks changed from 605.09 units to 3,411.59 units (463.82% increase), the outbreak duration changed from 16.77 days to 20.12 days (19.98% increase), and the number of outbreaks changed from 187 to 200 (6.95% increase).

### Sensitivity of the results from the culling strategy without routine vaccination to changes in the destruction ring radius

**Figure 5:**
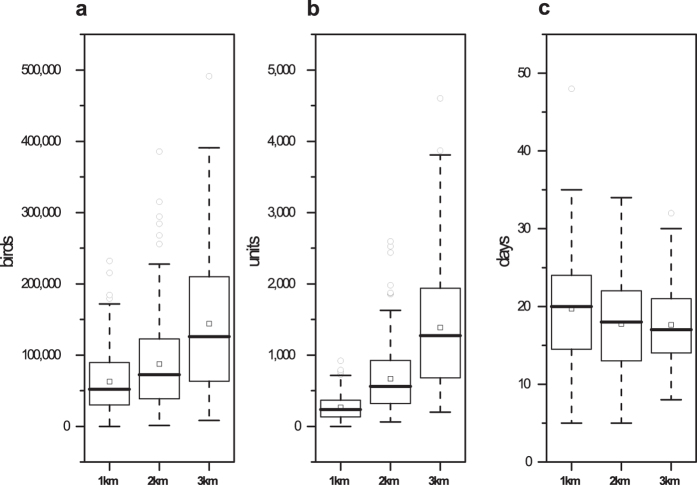
Results of the sensitivity analysis of changes in the destruction ring radius. The boxplots represent the number of depopulated birds, number of depopulated units and outbreak duration with a 1 km, 2 km and 3 km destruction ring radius. The squares (◽) represent the means under different conditions.

Fewer birds and flocks were depopulated but the outbreak duration was longer with the 1-km destruction ring than with the 3-km destruction ring. Suppose the radius of the destruction ring was changed to 1 km and 2 km; the results of 200 simulations are shown in Fig. 5. When the radius of the destruction ring was changed from 1 to 3 km (200% increase), the average number of depopulated chickens changed from 62,868 to 144,040 (129.11% increase), the number of depopulated flocks changed from 264.23 units to 1385.54 units (424.37% increase), the outbreak duration changed from 19.71 days to 17.63 days (10.55% decrease), and the number of outbreaks was unchanged (200 outbreaks).

### Direct costs

In this study, the control costs of an outbreak are the sum of the surveillance costs in the zones and the destruction costs. The control costs of the two different strategies (200 stochastic iterations) are shown in Table 1.

In this study, the direct cost of a culling strategy without vaccination equalled the control costs, and the direct cost of a culling strategy with routine vaccination equalled the sum of the control costs, vaccines and vaccination costs. To compare the direct costs of the two different strategies from 2004 to 2012, we assumed that the vaccine and vaccination cost of China’s layers and broilers was fixed at 5.15 billion^6^. The domestic poultry population in China has been massively vaccinated against H5N1 since late 2005. In 2005, epidemiological studies indicated that all prior outbreaks in China occurred in farms that did not vaccinate or vaccinated with unqualified vaccines^12^. In this study, we assumed that a culling strategy without vaccination was implemented from 2004 to 2005 and that a culling strategy with routine vaccination was implemented and used starting in 2006. The probability of an outbreak occurring was 51.5% when a culling strategy with routine vaccination was employed and was 100% in the absence of vaccination based on the simulation. From 2004 to 2012, 50, 31, 19, 8, 12, 4, 0, 2, and 8 H5N1 HPAI outbreaks occurred in China according to China’s official veterinary bulletin. From 2004 to 2012, 134 outbreaks would have occurred if a culling strategy without vaccination had been implemented, and 69 outbreaks would have occurred if a culling strategy with vaccination had been implemented.

As shown in Table 2, the direct costs of culling strategies without vaccination are significantly lower than the direct costs of culling strategies with routine vaccination. From 2004 to 2012, the direct cost of the former was only 0.63 billion yuan, whereas the direct cost of the latter was 46.4 billion yuan; thus, the direct cost of the former was only 1.36% of the direct cost of the latter.

## Discussion

We found that the routine vaccination strategy for H5N1 HPAI in China had a substantial impact on decreasing the number of H5N1 outbreaks. The simulation results showed that under the routine vaccination strategy, when a flock was infected, the probability of a H5N1 HPAI outbreak at the national level in China was 51.5%. Because the H5N1 virus is highly infectious among poultry, the risk of an H5N1 outbreak remains high when a flock is infected, even though the rate of qualified antibodies against the H5N1 virus is approximately 90% with the routine vaccination strategy used in China.

Compared with culling strategies without vaccination, culling strategies with routine vaccination can markedly reduce an H5N1 epidemic’s severity and duration at the national level in China. When a flock is infected, the total number of birds in the depopulated flocks and the total number of depopulated flocks can be reduced by approximately 90%. Additionally, the outbreak duration can be reduced by one-fourth (Fig. 1). Culling strategies with routine vaccination would have reduced the depopulation of 168,980 flocks or 17,501,470 birds between 2004 and 2012 (Table 2).

A higher unit density indicates a higher probability of H5N1 HPAI outbreaks with the routine vaccination strategy. According to the sensitivity analysis, when the unit density is changed from 0.5- to 2-fold of the average flock density in China, the probability of H5N1 HPAI outbreaks is increased from 51.5% to 60%. The routine vaccination strategy is more suitable for a nation/region with a low poultry unit density. In a country like China with a very high poultry unit density, the routine H5N1 vaccination strategy would play a limited role in decreasing H5N1 outbreaks. Five countries/regions with routine AI vaccination programmes had high to very high national poultry densities^4^. Indeed, the routine vaccination strategy is not suitable for all countries with high national poultry densities. The routine H5N1 vaccination strategy had different impacts on H5N1 outbreaks in countries with the same national poultry density but different unit densities (higher unit densities indicate a lower unit size with the same poultry density). Instead, the routine H5N1 vaccination strategy is more suitable for a country with a high national poultry density and low unit densities (high unit size).

From a cost perspective, China’s routine vaccination strategy for H5N1 HPAI is not a good strategy. If the culling strategies without and with vaccination were implemented separately, the direct cost of the former would have been only 1.36% of the latter from 2004 to 2012, and the strategies without the vaccination of chickens would have saved 45.77 billion yuan from 2004 to 2012. If ducks, geese and other poultry had control and vaccination costs similar to those for chickens (the chicken inventory is approximately 80% of the total poultry inventory in China), approximately 57 billion yuan would have been saved for all poultry if a strategy without routine vaccination had been used in China from 2004 to 2012. In most years, H5N1 HPAI outbreaks in China have numbered fewer than 10 since 2004. Although the indirect costs of H5N1 epidemics in China were not available, we believe that they were very limited in most years compared with the vaccination costs. Therefore, China’s routine vaccination strategy for H5N1 HPAI was not economical in most years studied.

The model outcomes were sensitive to changes in the flock density, flock size and contact rates (Figs 2, 3 and 4). A lower flock density, smaller flock size and lower contact rates indicated a lower epidemic severity. By reducing the direct and indirect contact of poultry, greater movement control and enhanced surveillance in areas around the detected units can effectively reduce the epidemic severity after a unit is infected. Rapid diagnoses and the reduction of the destruction ring radius contribute to a reduction in the loss of poultry in China (Fig. 5).

China’s H5N1 HPAI routine vaccination strategy could facilitate the emergence of vaccine-resistant viruses. The routine vaccination strategy would lose efficacy with the emergence of vaccine-resistant strains^13^. In February 2006, respiratory disease and decreased egg production were noted in some layer farms in Shanxi Province; the chickens in these farms were vaccinated with H5-inactivated vaccines. This finding suggested the presence of a new genotype of H5N1 avian influenza in China. To control the outbreaks, more than 2,311,000 birds were depopulated^5^.

This study was subject to several limitations. First, the model outcomes of H5N1 HPAI outbreaks were unaffected by the realistic spatial distribution of units and the actual unit size but were affected by the average unit density and average unit size of each unit size. Assuming that the geolocation of the units of each unit size obeys a uniform distribution seems insufficient for a large country like China. There is a very marked spatial concentration of poultry production in China. The eastern region of the country has a much higher poultry density than the central and western regions in terms of the total population density and producers. For example, Shandong Province in eastern China has the highest density of broilers for slaughter, 13,592 broilers for slaughter/km^2^ per year; Hainan Province in eastern China has the highest broiler farm density, 18 broiler farms/km^2^ per year; and Qinghai Province in western China has the lowest broiler density, 0.12 broiler farms/km^2^ and 1.37 broilers for slaughter/km^2^ per year. Additionally, basic spatial structures (i.e., roads, cities, and markets) were not included in the model. However, the assumption that the geolocation of units of each unit size obeys a uniform distribution has its advantages. The aim of this study was to assess the routine H5N1 vaccination strategy at the national level but not at the regional level. If the location-specific poultry density and basic spatial structure were included in the model, the geolocation of the initial infected unit would have had a huge impact on the simulation results. Thus, the geolocation of the initial infected unit of each simulation would be very difficult to determine. Second, little evidence was available to provide well characterized parameter estimates, and we relied on limited information and expert opinion. However, this lack might not be a serious problem because this study emphasizes the comparison of the epidemiological and economic impacts of two H5N1 control strategies. Third, risk assessment of human infection with the H5N1 virus was not investigated in this study. Finally, estimating China’s H5N1 vaccination usage and coverage rates is difficult. Although the government requires 100% vaccine coverage in domestic poultry in China, there are a huge number of small-scale or backyard farms, and the goal of 100% vaccination is impossible to realize. Chen estimated the annual H5 AI vaccine usage was 11.5 to 13.2 billion doses per year from 2006 to 2008^5^. Swayne estimated that the annual H5 AI vaccine usage was 18.9 to 23.7 billion doses per year from 2006 to 2009^4^. The rate of qualified antibodies against the H5N1 virus in China was approximately 90% in recent years according to China’s official veterinary bulletin, indicating that China’s H5N1 vaccination coverage rate was above 90%. In this study, we supposed that culling strategies without vaccination were implemented in China from 2004 to 2005. A total of 78 H5N1 HPAI outbreaks occurred and 11,657,000 birds were depopulated in China from 2004 to 2005 (four outbreaks that occurred in 2005 in Liaoning province were not included because these outbreaks were confirmed at least two weeks after the disease was found by local farmers; the delayed disease reporting resulted in 19,959,000 birds being depopulated^5^). The mean number of depopulated birds with culling strategies without vaccination was 149,449 during an outbreak in China from 2004 to 2005, which was very close to the simulation result of 144,040.

The routine vaccination programme has been effective and has played an important role in reducing H5N1 outbreaks and markedly reducing the numbers of depopulated birds in China. However, the H5N1 outbreaks were not eradicated by the routine vaccination programme; this issue combined with the high costs of vaccines and vaccination facilitated the emergence of vaccine-resistant viruses and reduced the efficacy with the emergence of vaccine-resistant strains. Thus, China should exit the H5N1 routine vaccination programme at the national level. Traditional H5N1 HPAI control strategies based on culling programmes would be preferable at the national level in China.

## Methods

### Epidemiological model

The North American Animal Disease Spread Model (NAADSM) was used in this study. The NAADSM is a spatially explicit, stochastic, state-transition model for the spread of highly contagious animal diseases that can be used to simulate disease control strategies and estimate the epidemiological and economic impacts of these strategies^14^. Wei simulated H5N1 control strategies for a county in China and estimated the epidemiological and economic impacts of these strategies using NAADSM 3.1^6^. Patyk simulated the spread and control of highly pathogenic avian influenza (H5N1) among commercial and backyard poultry flocks in South Carolina using NAADSM 3.2^11^.

### Data and control strategies

Chickens represent the largest poultry population in China, with the chicken inventory accounting for more than 80% of the total poultry inventory. Chickens are also the species in which H5N1 HPAI occurs most frequently in China. China had 787 million ducks and 325 million geese in 2010, but data on the flock densities and sizes of China’s duck and geese populations were not available. Thus, duck and geese data were not included in simulation.

Disease spread in this study was simulated at the unit level (i.e., at the flock level) rather than at the level of an individual animal. For the purposes of this study, each commercial and backyard flock was considered a single unit. Each unit in this study was characterized by its production type, unit size (the number of animals in the unit) and geolocation specified by latitudinal and longitudinal coordinates. The production types were assigned based on groups of units with similar species, unit sizes, disease transmission probabilities, disease manifestations, disease detection probabilities, and control strategies^11^.

Data regarding the geolocations and unit sizes of chickens in China are not available. There are 14 unit sizes, including 7 unit sizes for layer inventory (1–499, 500–1999, 2000–9999, 10000–49999, 50000–99000, 100000–499999 and above 500000) and 7 unit sizes for broilers for slaughter (1–1999, 2000–9999, 10000–49999, 50000–99999, 100000–499999, 500000–999999 and above 1000000), in the “2011 China Animal Industry Yearbook” (since 2012, the average unit size of China’s layers and broilers were not available in the “China Animal Industry Yearbook”). In this study, we assumed that the geolocation of each unit size obeyed a uniform distribution. The unit density of a given unit size was calculated using the number of units of this size divided by China’s area. The geolocation of a unit with a given size was created at random in a location according to its unit density on China’s map. Only chickens in mainland China were included in the study. Two control strategies were evaluated:

1. Culling strategy with routine vaccination: This strategy is a routine vaccination programme for all poultry; poultry within a 3-km radius of a detected infected unit are killed, and an 8-km “moderate risk” zone is placed around detected infected units. Measures are used to restrict disease spread within this “moderate risk” zone.
2. Culling strategy without vaccination: The same activities described for the culling strategy with routine vaccination are employed except that no vaccination programme is included.

### Input parameters

The input parameters include the following: animal population, disease, disease spread, disease detection, tracing, zones, destruction, vaccination and cost accounting. The values for the parameters in the model were determined based on a review of the literature^6^,^11^ and expert opinions.

1 Animal populations

(1) Production type

The following four production types were considered: layer units of 1 to 499 birds (production type 1), layer units with more than 500 birds (production type 2), units of broilers for slaughter ranging from 1 to 1999 birds per year (production type 3) and units of broilers for slaughter with more than 2000 birds per year (production type 4). Small-scale chicken producers still prevail in China. Thus, we assumed that a unit of production type 1 was infected during the simulation. In each simulation, we assumed that a unit of production type 1 was in the latent state and other flocks were in the susceptible state.

(2) Unit size

The unit size of a chicken unit is equal to the average chicken inventory of the unit size (e.g., the broiler inventory is one-quarter of the broilers for slaughter) in the “2011 China Animal Industry Yearbook”.

2. Disease

Four H5N1 disease states were included in the study: latent, subclinical, clinical and immune. The parameters latent period duration, infectious subclinical period, infectious clinical period and immune period were as follows: Latent: the time parameters are 0, 1, 2, 3, and 7 days, and the probability density function parameters are 0, 0.35, 0.35, 0.12, and 0, respectively; thus, on days 0, 1, 2, 3 and 7, the probability density functions values are 0, 0.35, 0.35, 0.12, and 0, respectively. Subclinical: the time parameters are 0, 1, 2, 3, and 8 days, and the probability density function parameters are 0, 0.15, 0.5, 0.12, and 0, respectively. Clinical: the time parameters are 0, 2, 4, 6, and 10 days, and the probability density function parameters are 0, 0.07, 0.28, 0.1, and 0, respectively. Immune: following a triangular distribution, the parameters are 10, 21, and 120^14^.

These disease states apply to the entire unit rather than to individual birds. The probability that infected units will die from disease is 95%.

3. Disease spread

Parameters were established for disease transmission by direct contact and indirect contact. In the present study, direct contact involved chickens in a source unit coming into contact with chickens in a recipient unit. In contrast, indirect contact included contacts involving the movement of people, vehicles, equipment, materials, and animal products between units that could result in disease transmission. Because most commercial operations practise all-in, all-out management on the premises, there are few opportunities for direct contact. However, the potential risk of direct contact does exist among backyard poultry (e.g., by acquisition of new birds or bird movement between neighbouring premises)^10^. Latently infected units were assumed to be capable of transmitting infection to other units only by direct contact. Clinically infected units were assumed to be capable of transmitting infection to other units by both direct and indirect contact.

In the present study, four parameters were used to define disease transmission by contact: the contact rate (i.e., the mean number of contacts generated by each unit on each day), probabilities of infection transfer, contact distances and effects of movement restrictions.

(1) Contact rate and probabilities of infection transfer

The contact rates and probabilities of infection transfer of the different production types based on the literature^6^,^11^,^15^ and expert opinions are listed in Table 3.

Because the rate of qualified antibodies against the H5N1 virus in China was approximately 90% in recent years according to China’s official veterinary bulletin, we assumed that the probabilities of infection transfer in the culling strategy with routine vaccination were 10% of those of the culling strategy without vaccination.

(2) Contact distances

Contact distances are determined in part using a probability density function. In this study, there were three contact distance types: near distance, middle distance and far distance. All of the types followed a triangular distribution, with near distance parameters of 0.08, 0.3, and 1.6 km (i.e., the minimum distance is 0.08 km, the maximum distance is 1.6 km and the most likely distance is 0.3 km), middle distance parameters of 1, 1.5, and 10 km, and far distance parameters of 1, 3, and 20 km. We assumed that the contact distance of small-scale producers was near distance and the contact distance of large-scale producers was far distance. If a small-scale producer is the source of one exposure and a large-scale producer is the recipient, the contact distance is a far distance. If a large-scale producer is the source of one exposure and a small-scale producer is the recipient, the contact distance is a middle distance.

(3) Movement restrictions

After the detection of disease, movement restrictions are used to reduce the number of contacts between units, thereby reducing the possibility of disease spread. For this study, we assumed that the contact rate was reduced to 60% of the normal rate on the day that the disease was detected; the contact rate was reduced to 30% on the third day after detection and 20% on the fifth day after detection.

4. Disease detection

There are two probabilities that an infected unit will be detected: the probability that clinical signs will be observed in an infected unit and the probability that a unit will be reported once clinical signs have been observed.

The probabilities of observing clinical signs are 50%, 72% and 90% on the 1st, 2nd and 3rd days of a herd’s clinical period. The probabilities of reporting units with observed clinical signs are 10%, 60% and 90% on days 0, 5, and 10 after the disease is first detected.

5. Tracing

Tracing refers to the process of identifying units at high risk for disease based on contact with detected units. Trace-forward investigations of direct and indirect contacts were conducted for all production types in this study. Traces were initiated upon the detection of an infected unit, and all traces were conducted for all units that had contact with the detected unit in the 5 days prior to detection. The probabilities of trace success for both direct contact and indirect contact are 0.9 and 0.7, respectively.

6. Zones

The detection of infected units from clinical signs will result in the placement of a zone around the unit. Zones are used to restrict disease spread by direct and indirect contact among units. We used a 3-km “high risk” zone and a 3- to 8-km radius “moderate risk” zone in this study. The direct movement rates in a “high risk” zone are reduced to 50%, 30% and 10% on days 0, 1 and 2 after a new zone focus is created. The direct movement rates in a “moderate risk” zone are reduced to 60%, 30% and 10% on days 0, 1 and 2 after a new zone focus is created. After a new zone focus is created, the multiplier for the probability of observing clinical signs in a zone is 1.2.

7. Destruction

We assumed that the delay before implementing destruction was one day after the first detection of an infected unit and that the destruction capacity reached its maximum after 3 days. On days 0, 1, 2, 3, and 10 after the detection of disease, the destruction capacities were 5000, 10000, 15000, 30000 and 30000 units per day, respectively. Destruction capacities are quite high in China. In October 2005, over 6 million birds were depopulated in three days in Liaoning province.

The radius of the destruction ring around an infected unit is 3 km. If the destruction capacity is limited, the destruction priorities are as follows: production type 4, production type 2, production type 3 and production type 1.

8. Cost accounting

The control costs during an outbreak period include:

(1) Costs of surveillance zones

The cost of surveillance per animal per day associated with a “high risk” zone is 1.5 yuan, and the cost of surveillance per animal per day associated with a “moderate risk” zone is 0.5 yuan.

(2) Costs of destruction

The cost of indemnification is 20 yuan per bird, the cost of euthanasia is 5 yuan per bird, and the cost of carcass disposal is 1 yuan per bird. The cost associated with the appraisal, cleaning and disinfection of each destroyed backyard poultry unit is 200 yuan, and the cost of each destroyed commercial unit is 700 yuan.

## Additional Information

**How to cite this article**: Sun, Z. *et al*. Assessment of China’s H5N1 routine vaccination strategy. *Sci. Rep.* **7**, 46441; doi: 10.1038/srep46441 (2017).

**Publisher's note:** Springer Nature remains neutral with regard to jurisdictional claims in published maps and institutional affiliations.

## Figures and Tables

**Table 1:** H5N1 HPAI control costs of the two different strategies in China.

Strategy	Variable	Mean	Std Dev	Low	High
Destruction without vaccination	Surveillance costs in zones (thousand yuan)	950.5	767	112.2	4052.6
	Destruction costs (thousand yuan)	3730.8	2530.9	217.3	12556
	Control costs (thousand yuan)	4681.3	—	—	—
Destruction with vaccination	Surveillance costs in zones (thousand yuan)	54.4	105.9	0	923.9
	Destruction costs (thousand yuan)	350.6	467.1	0	2357.2
	Control costs (thousand yuan)	405	—	—	—

**Table 2:** The number of depopulated birds, the number of depopulated units and direct costs of the two different strategies in China from 2004 to 2012.

Strategy	Variable	
Destruction without vaccination	Total number of birds in depopulated flocks (thousands)	19,301.36
	Total number of depopulated flocks (thousands)	185.66
	Control cost (billion yuan)	0.63
	Direct cost (billion yuan)	0.63
Vaccination plus destruction	Total number of birds in depopulated flocks (thousands)	1,799.89
	Total number of depopulated flocks (thousands)	16.68
	Control cost (billion yuan)	0.05
	Vaccination cost (billion yuan)	46.35
	Direct cost (billion yuan)	46.4

**Table 3:** Contact rates between production types and probabilities of infection transfer.

Sources and recipients	Disease spread	Contact rate	Probability of infection given contact
type 1 → type 1	Direct contact, indirect contact	1.2	0.25
type 1 → type 2	Indirect contact	0.5	0.4
type 1 → type 3	Direct contact, indirect contact	1.2	0.25
type 1 → type 4	Indirect contact	0.2	0.3
type 2 → type 1	Indirect contact	0.5	0.25
type 2 → type 2	Indirect contact	0.9	0.4
type 2 → type 3	Indirect contact	0.2	0.25
type 2 → type 4	Indirect contact	0.45	0.3
type 3 → type 1	Direct contact, indirect contact	1.2	0.25
type 3 → type 2	Indirect contact	0.4	0.3
type 3 → type 3	Direct contact, indirect contact	1.2	0.25
type 3 → type 4	Indirect contact	0.6	0.4
type 4 → type 1	Indirect contact	0.2	0.25
type 4 → type 2	Indirect contact	0.26	0.3
type 4 → type 3	Indirect contact	0.26	0.25
type 4 → type 4	Indirect contact	0.4	0.4
